# A Case of Idiopathic Hypereosinophilic Syndrome Causing Mitral Valve Papillary Muscle Rupture

**DOI:** 10.1155/2015/538762

**Published:** 2015-11-12

**Authors:** Tiffany Tamse, Avind Rampersad, Alejandro Jordan-Villegas, Jill Ireland

**Affiliations:** ^1^Florida Hospital for Children, Orlando, FL 32803, USA; ^2^University of Central Florida, Orlando, FL 32827, USA; ^3^Orlando Health, Orlando, FL 32806, USA

## Abstract

Idiopathic Hypereosinophilic Syndrome (IHES) is a rare disease that can be difficult to diagnose as the differential is broad. This disease can cause significant morbidity and mortality if left untreated. Our patient is a 17-year-old adolescent female who presented with nonspecific symptoms of abdominal pain and malaise. She was incidentally found to have hypereosinophilia of 16,000 on complete blood count and nonspecific colitis and pulmonary edema on computed tomography. She went into cardiogenic shock due to papillary rupture of her mitral valve requiring extreme life support measures including intubation and extracorporal membrane oxygenation (ECMO) as well as mitral valve replacement. Pathology of the valve showed eosinophilic infiltration as the underlying etiology. The patient was diagnosed with IHES after the exclusion of infectious, rheumatologic, and oncologic causes. She was treated with steroids with improvement of her symptoms and scheduled for close follow-up. In general patients with IHES that have cardiac involvement have poorer prognoses.

## 1. Introduction

Hypereosinophilic Syndrome (HES) is a rare disease in childhood, usually occurring between 20 and 50 years of age, with the true prevalence unknown [1]. The Idiopathic HES variant is even less common. Cardiovascular involvement in IHES is not uncommon and poses the highest risk for morbidity and mortality. This case report of a 17-year-old female describes a course of cardiogenic shock resulting from an acute papillary muscle rupture secondary to eosinophilic infiltration. No cases have been reported in the pediatric literature and very few have been reported in the adult literature of papillary rupture [2]. It is important to recognize that, despite being a rare occurrence, marked hypereosinophilia in a child should be worked up thoroughly at presentation.

## 2. Case Report

This is a 17-year-old female with a history of depression, anxiety, chronic abdominal pain, and heavy and painful menses for which she takes an oral contraceptive pill. She presented with a two-week history of worsening abdominal pain, described as a constant dull ache located in the pelvic region and accompanied by nausea with bilious emesis, and mild chest pain. She had been seen in her primary care physician's office four days prior to admission and given hyoscyamine for her pain with minimal relief. No associated menstrual symptoms were noted.

The patient admits to smoking 5–7 cigarettes daily but denies any drug or alcohol use. Her last sexual encounter was 6 months prior to admission and she denies any prior pregnancy or sexually transmitted infections.

She presented to the Emergency Department and vital signs showed tachycardia and elevated blood pressure. Her cardiac examination was normal and there was no prior history of any murmur. Her abdominal exam showed diffuse tenderness in the lower quadrants and pelvic area, without distension. Cervical motion tenderness was elicited and white discharge was noted on pelvic exam. Wet prep was negative for trichomonas, yeast, and clue cells. Laboratory values were significant for leukocytosis of 30,850 and eosinophilia of 52.4%. Computed tomography (CT) of the abdomen showed thickening and edema of the wall of the duodenum and proximal jejunum, ascites in the upper abdomen, and appearance of a collapsed hemorrhagic cyst/corpus luteum in the left adnexa. The patient was given intravenous doxycycline, cefotetan, and analgesia prior to admission.

Gynecology was consulted and treatment was directed towards a ruptured ovarian cyst. No surgical intervention was recommended and plan was to treat patient for a possible culture negative pelvic inflammatory disease. Repeat complete blood count was unchanged with eosinophilia and leukocytosis and clinically patient was improving.

On day two of admission she had an acute decompensation with bilious emesis and severe hypotension. She became hypoxic and was transferred to the pediatric intensive care unit, for suspected septic shock, where she was emergently intubated and started on inotropic support. She was taken to the operating room for an exploratory laparotomy. The small bowel was edematous and thickened throughout the proximal jejunum with full thickness enteritis. Areas of serosal erosion and adherent omentum as well as an area of pale bowel with lymphadenopathy were noted. A diverting jejunostomy was performed to allow the inflamed bowel to decompress and a specimen was sent to pathology.

**Figure 1:**
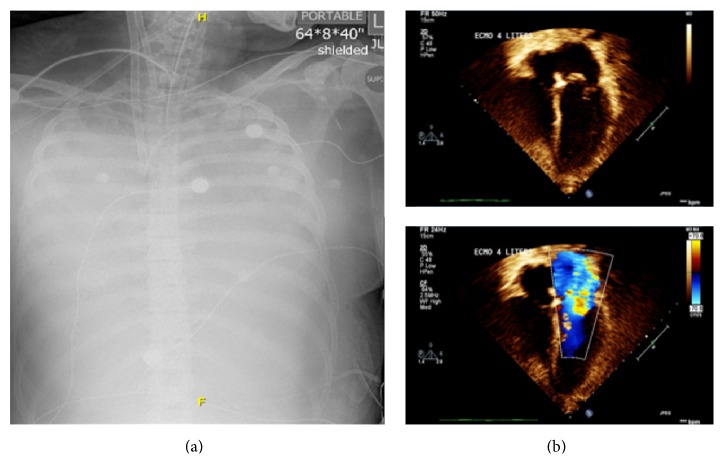
(a) Chest X-ray showing complete opacification of the thorax suspicion for complete lung consolidation secondary to edema. (b) Echocardiogram images showing flail mitral valve from a ruptured cord and severe mitral regurgitation.

She continued to decompensate and was started on high frequency oscillatory ventilation with nitric oxide. Her chest X-ray showed worsening interstitial and alveolar edema (Figure 1(a)). At this time the patient went into multiorgan dysfunction syndrome, due to increased level of support, she was placed on VA ECMO. Cardiac echocardiography demonstrated severe mitral regurgitation from a nonfunctioning valve, moderate left atrial enlargement (Figure 1(b)). The patient was taken to the operating room for replacement of the mitral valve and intraoperatively there was noted to be papillary muscle avulsion of the anterior leaflet without evidence of vegetation. She was separated from ECMO support but developed acute renal failure requiring one week of hemodialysis. She was extubated on day ten of hospitalization and kidney function recovered completely. Neurologically she has remained without any deficits noted.

**Figure 2:**
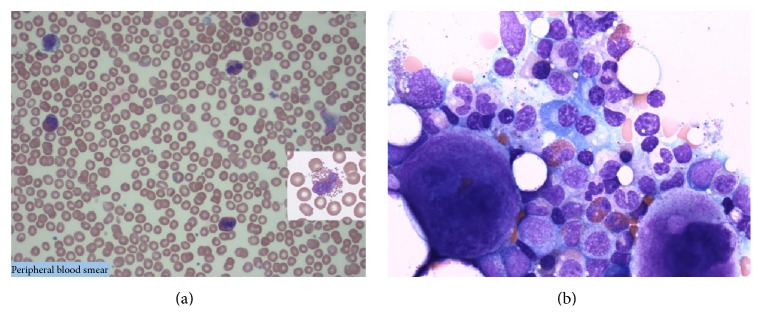
(a) Peripheral smear: marked eosinophilia (most eosinophils show intact cytoplasmic granules). (b) Bone marrow biopsy: normocellular marrow with mild megakaryocytic hyperplasia, eosinophilia, and no increase in blasts. No monoclonal B-cells or immunophenotypically abnormal T-cells are detected.

**Figure 3:**
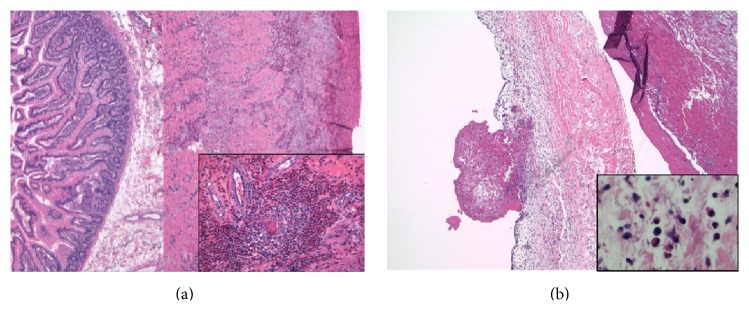
(a) Jejunum: diffuse marked eosinophilic infiltrate associated with ischemic tissue damage. (b) Mitral valve: benign endomyocardial tissue demonstrating myocardial necrosis and fibrinous endocarditis with mixed inflammatory infiltrate comprising of eosinophils, lymphocytes, and histiocytes.

Pathology specimens, peripheral smear, bone marrow, bowel, and heart valve all demonstrated marked eosinophilic infiltration (Figures 2 and 3). Workup was negative for vasculitis, collagen vascular, infectious, and myeloproliferative diseases (Table 1).

She was diagnosed with IHES and received a single dose of ivermectin before she was started on solumedrol. She was discharged to inpatient rehabilitation on day 27 and then returned for ostomy reversal 5 days later.

## 3. Discussion

HES is defined as an absolute eosinophil count of greater than 1,500/mm^3^ that is associated with end organ damage that cannot be explained by another cause aside from the eosinophilia [3]. Historically there was a requirement of 6 months of hypereosinophilia but this is no longer the case as patients have presented with significant end organ damage warranting the diagnosis and treatment [3, 4].

HES among the pediatric population has a heterogeneous presentation, most commonly with fever, arthralgias, and rash [5]. Other nonspecific symptoms include fatigue, cough, dyspnea, and rhinitis. The disease can affect about every organ system through eosinophilic infiltration of the tissue causing varying degrees of damage and can be life threatening [4]. The major basic proteins in the cells are thought to play a role in the pathogenesis of this disease [6].

IHES, which we believe our patient has, is a diagnosis of exclusion and can only be made once all secondary causes of hypereosinophilia have been excluded. There must be an absence of eosinophil blasts in the blood and bone marrow [7].

There may be a different underlying pathogenesis in IHES of pediatric patients as compared to adult patients. There is only a slight male predominance in the pediatric population, whereas adult males have a predominance of 9 : 1 [5]. Also although chromosomal abnormalities and acute leukemia account for many pediatric cases for HES, rarely the FLP1L1-PDGFRA fusion gene is found, which is commonly seen in adults [1].

Cardiac involvement carries the highest morbidity and mortality and classically occurs in stages. Degranulated eosinophils in peripheral blood smears can indicate endocardial disease [6]. Eosinophils infiltrate the tissue and release toxic mediators. This causes endocardial damage and platelet thrombi leading to mural thrombi and risk for emboli. Over time fibrous thickening of the endocardial lining leads to a restrictive cardiomyopathy. The tricuspid and mitral valves can be effected leading to regurgitation [3]. Our patient had evidence of fibrinous endocarditis with mixed inflammatory infiltrate of eosinophils, lymphocytes, and histiocytes. There was also intravascular debris demonstrating partially degenerated eosinophils.

Eosinophilic colitis with low ESR, as was present in our patient, is a common GI manifestation, as well as hepatitis, hepatosplenomegaly, cholangitis, and pancreatitis [7, 8]. Pathologic investigation of the bowel sample from our patient demonstrated diffuse marked eosinophilic infiltration associated with ischemic tissue damage and an ill-defined granulomatous reaction. Additionally, there was focal fibrinous necrosis of the small vessels as well as intravascular thrombi and eosinophilic perivasculitis.

Neurologically the disease can present as hemiplegia (focal deficits), peripheral neuropathy, or an altered behavior or cognitive dysfunction. Most patients with IHES with neurologic involvement also have an associated endocarditis with emboli being a source of the CNS complications. Neurotoxins have also been found in eosinophils which may account for the diffuse altered mental status [9].

Dermatologic manifestations include mucocutaneous ulcers, pruritic papules, and nodules as well as a reported case of recurrent vesicular rash with necrotic crusting lesions [10]. Pulmonary presentation includes chronic cough, pulmonary infiltrates, fibrosis, and embolus. Hematologic insults including idiopathic thrombocytopenic purpura have been reported [4].

The differential for causes of hypereosinophilia is vast and a thorough investigation is indicated [6]. Workup for IHES should follow serial eosinophil counts. Blood and stool studies should be done to exclude parasitic infections. Peripheral smear initially and then bone marrow biopsy should be performed to rule out a malignancy. The vasculitides may be ruled out by ANCA, ANA, complements, ESR, and RF. HIV and Bartonella serology may be performed to rule out these diseases if at risk. Chest X-ray and CT of chest and abdomen may reveal pathology. At persistent eosinophil levels above 1,500, an ECHO must be performed and followed. Troponins may also be helpful in detecting asymptomatic endocarditis from eosinophilic infiltration [8].

The goal of treatment is to keep absolute eosinophil counts below 1,500. If patients are asymptomatic they may be observed but with serial echocardiograms and close follow-up. First-line treatment for IHES is corticosteroids [11]. Our patient responded to a 2-week methylprednisolone course with return of her eosinophils to below 500. Duration of therapy depends on response and control of symptoms and the steroids are tapered to the lowest efficacious dose. Some patients may need lifetime treatment. Interferon alpha, which inhibits proliferation of eosinophil precursors, is 2nd line for nonresponders to steroids. Hydroxyurea, vincristine, and other chemotherapeutic drugs are 3rd line [8]. Imatinib has primary use in patients with PDGFRA/B mutations but has also been useful even when no mutation is found, although this may be due to multiple variations of the mutations that may not have been detected [3]. Mepolizumab has been used for the FIP1L1-PDGFRA mutations as well. Bone marrow and peripheral stem cell transplantation for refractory causes is under investigation.

Surgery including heart valve replacement for severe mitral regurgitation is not uncommon although it is usually not as emergent as in our patient whose regurgitation was caused by papillary muscle rupture. Endocardial decortication and heart transplant may also be warranted but not as commonly [12]. Splenectomy for the disease has also been reported [3].

If untreated, the morbidity and mortality can reach 80% at 3 years for the IHES. With treatment the survival rate increases to 80% at 5 years and 42–60% at 10 years [8]. Poor prognostic factors include cardiac involvement, higher WBC count at presentation, and myelodysplastic features, while patients with good steroid response and elevated IgE levels have a better prognosis.

## Figures and Tables

**Table 1:** 

*Infectious*	
HIV 1/2 Ag/Ab	Nonreactive
Hepatitis A, hepatitis B, and hepatitis C	Nonreactive
*Bartonella henselae* PCR	Negative
*Toxocara*	Negative
*Strongyloides*	Negative
*Tropheryma whipplei* PCR	Negative
Blood/fungus cultures	Negative
Urine cultures	Negative
*Immunologic *	
ANA (blood and abdominal fluid)	Negative
DSDNA/SSA Ab	Negative
Smith/RNP Ab	Negative
ANCA	Negative
C3	39 mg/dL
C4	2 mg/dL
IgG	345 mg/dL
IgA	111 mg/dL
IgM	143 mg/dL
IgE	191 kU/L
CD4 helper cells	34.3%
CD8 suppressor cells	26.4%
Total CD3	66.2%
Total B-cells	28.9%
Natural killer cells	3.2%
CD4/CD8 ratio	1.3
T-cell interpretation	Normal total B-cells (CD19)
*Other *	
Beta hCG (urine)	Negative
Cortisol	13.4 *μ*g/dL
Tryptase	2.6 *μ*g/L
*Genetic analysis*	
PDGFRa (4q12) FISH	Normal
PDGFRb (5q33) FISH	Normal
FGFR1 (8p11) FISH	Normal
BCR/ABL1/ASS1 t(9,22) FISH	Normal
KIT (c-KIT) mutation	Not detected
Next-Gen Sequencing myeloid disorders profile^*∗*^ (NeoTYPE analysis)	No evidence of mutation in any of genes tested. Low probability of myeloid neoplasm diagnosis (<10%)

^*∗*^Myeloid disorders profile: ABL1, ASXL1, ATRX, BCOR, BCORL1, BRAF, CALR, CBL, CBLB, CBLC, CDKN 2A, CEBPA, CSF3R, CUX1, DNMT3a, ETV6, EZH2, FBXW7, FLT3, GATA1, GATA2, GNAS, HRAS, IDH1, IDH2, IKZF1, JAK2 Exon 12 + 14, JAK3, KDM6A, KIT, KRAS, MLL, MPL, MYD 88, NOTCH 1, NPM1, NRAS, PDGFRA, PHF 6, PTEN, PTPN11, RAD21, RUNX1, SETBP1, SF3B1, SMC1A, SMC3, SRSF2, STAG2, TET2, TP53, U2AF1, WT1, and ZRSR2.

## Abbreviations

IHES: Idiopathic Hypereosinophilic Syndrome
HES: Hypereosinophilic Syndrome
ECMO: Extracorporal membrane oxygenation.
